# Visual Impairment, Inclusion and Citizenship in South Africa

**DOI:** 10.5334/aogh.4245

**Published:** 2024-04-05

**Authors:** Brian Watermeyer, Michelle Botha, Heidi Lourens, Xanthe Hunt

**Affiliations:** 1 Centre for Disability and Rehabilitation Studies, Department of Global Health, Faculty of Medicine and Health Science, Stellenbosch University, South Africa; 2 Department of Psychology, University of Johannesburg, South Africa; 3 Institute for Life Course Health Research, Department of Global Health, Faculty of Medicine and Health Science, Stellenbosch University, South Africa

**Keywords:** Visual impairment, inclusion, citizenship, education, rehabilitation

## Abstract

People with visual impairment face significant material challenges to access and inclusion in South Africa. These are in large part rooted in and supported by prejudiced assumptions about the needs, nature and capabilities of this group. The cultural and psychological face of oppression needs to be attended to. To this end, this viewpoint brings together the work of three visually impaired scholars in three key areas pertaining to the promotion of the inclusion and citizenship of visually impaired persons in South Africa. These areas are education; rehabilitation; and social inclusion and visibility. This work argues that undoing lifelong exclusion requires examining how disablism is embedded in the very fabric of our societies and operational at various levels: material, administrative, cultural and relational.

## Introduction

Around the world, disabled people remain an oppressed, marginalised and disadvantaged minority, with unequal access to essential services and resources, such as health care, education, appropriate housing, transportation, and employment [1]. In low- and middle-income (LMI) contexts, including South Africa, disability interacts with poverty in a vicious cycle, compounding deprivation [2]. Persons living with visual impairment (VI) are no exception. Unemployment among South Africa’s 724,000 persons with visual impairment is estimated at 97% [3]. Besides these alarming material realities, the oppression of visually impaired persons also has a cultural and psychological face, with prejudiced assumptions about the human potential of this group being almost ubiquitous [4,5,6]. Many visually impaired persons grow up surrounded by myths and stereotypes about damage, dependency and incapacity, which may become internalised, leading to further exclusion [7].

This paper presents research material in three domains (involving contributions from the first three authors), each of which is a key area of response to the social oppression of visually impaired persons in our society. Firstly, we address the burning issue of access to education, discussing the question of how best to prepare school teachers for the full and equitable inclusion of learners with VI in their classrooms. Secondly, we approach the issue of rehabilitation and empowerment. Rehabilitation for visually impaired persons has been widely criticised for failing to address the internalised aspects of disability oppression, with implications for the creation of a community more able to claim its legitimate citizenship rights. Lastly, we turn to the issue of how the experience of visually impaired persons, and of disabled persons in general, has been silenced across the world, with implications for mental health as well as political visibility. Auto-ethnographic writing that explores the phenomenology of VI in social contexts is put forward as one means of addressing this invisibility.

## 1. Education (Brian Watermeyer)

### Disability as ‘matter out of place’: Emotional issues in inclusive education for learners with visual impairment

In 1994, representatives from 92 countries attended the World Conference on Special Needs Education in Salamanca, Spain and agreed upon a global policy directive, which became known as the Salamanca Statement [8]. This cemented the position that children with disabilities should be educated in inclusive settings along with their non-disabled peers, a key principle for the provision of equitable education. Since this statement, there has been a global turn towards educating children with disabilities in inclusive settings, rather than segregated special schools. Consequently, there is a worldwide need to upskill teachers for educating learners with a range of impairments. But besides a lack of skills, teachers also carry anxieties and prejudices about disability difference, which need addressing, such as low expectations and negative cultural associations [9]. Teachers are products of societies where disability has been managed through segregation, supporting stereotypes of low potential and scepticism about educational inclusion.

South Africa’s inclusive education policy, Education White Paper 6 [10] has been poorly implemented, contributing to a national crisis involving the mass exclusion of disabled children from basic education [11]. The skills deficit is compounded by systemic problems across the education system, including inadequate infrastructure, large class sizes, poor school leadership and corruption.

A three-year project (2016–2019) funded by the EU and CBM (Christoffel-Blindenmission - an international disability development NGO) responded by developing training courses for practicing teachers, focusing on educating learners with sensory and intellectual impairments [12]. As a leader of the team developing the course on VI, I faced the challenge of deciding what to fit into a mere 30 hours of contact teaching, with instrumental skills (such as teaching methods, inclusive classroom design and curriculum adaptation) jostling with relational skills training aimed at addressing disability-related anxieties and prejudices.

Evidence suggests that most teachers have little understanding of disability as an issue of social justice, and that theoretical tuition in this area does not shift oppressive ‘medical model’ views — in other words, positions that ignore the contextual aspects of disability oppression in favour of an individualising account focusing purely on disease [13]. The ‘hard’ instrumental skills mentioned above are essential, technical elements of providing curriculum access to learners with VI. However, these have historically sidelined relational skills, which address emotional barriers to accepting, secure relationships with disabled children, regarded as an essential basis for learning [14]. Empathy, good listening and self-awareness facilitate open communication, which is essential for identifying and addressing barriers to access. Developing these skills requires training that is not just cognitive, but also brings emotional shifts at the level of one’s relationship to disability. Broderick and Lalvani [13] call for ‘experiential learning that engages deeply with personal experience and emotion’, a type of training that is self-reflective and transformative.

We used ideas from critical psychoanalysis, which views discrimination as emanating from how disability appeals in the observer to universal existential anxieties. These anxieties relate to, among other things, the frailty and impermanence of the body, inadequacy and dependence, and mortality [15]. Defence against these unwanted feelings manifests in impulses to distance, control, subdue and conceal disabled persons across society, rendering systemic exclusion, deprivation and abuse. Difficult emotions also work at the interpersonal level leading to anxiety, which disrupts authentic relations and creates barriers between persons with and without disabilities [16]. VI in particular brings forth negative and fearful cultural associations to do with ideas of indignity and loss of control, the destruction of agency and shame-ridden myths of divine punishment [17].

Our data showed that the ‘new unknown’ of visually impaired learners in the classroom brought about difficult emotional reverberations to already overloaded teachers, disrupting the optimism and creativity essential for solving access problems in low-resource environments. In our conceptualisation, the teachers we worked with experienced the presence, emotions and needs of learners with VI as ‘matter out of place’ — in other words, as an anomaly or ‘excess’, a perplexing threat to the social order. Anthropologist Mary Douglas [18] described how such ‘anomalies’, or threats to the prevailing culture, may be responded to in controlling ways by the community, aiding our understanding of the psychosocial dynamics at play.

We reasoned that if teachers were to be able to accept the lived realities and emotions of visually impaired learners, they first required an experience of their own difficult feelings being ‘contained’ [19]. In our short course, we added daily open-ended, reflective sessions led by a team of four highly educated visually impaired persons (thereby upending disablist stereotypes), who had experiences both of inclusive and special education settings. Teachers were encouraged to share their feelings in a non-judgmental environment, where ‘political incorrectness’ was welcomed. The panel gently assisted the teachers in making sense of ambivalence and talking through anxious fantasies about managing inclusion. Through the panel members’ own stories of schooling and a strategic use of humour, ‘unspeakable’ truths about life with VI could be discussed, defusing the power of what, in disability, so often remains unsaid [9]. The discussions were framed as the outset of a process of self-engagement regarding feelings about disability, over time opening the way for empathic relating, and the ability to listen without othering. Qualitative data gathered six months after the training reflected substantial, encouraging shifts in teachers’ feelings and motivations surrounding the inclusion of visually impaired learners [10].

## 2. Rehabilitation (Michelle Botha)

### Critique of rehabilitation services for persons with visual impairment in South Africa

Globally, policies that underpin the provision of rehabilitation services for persons with congenital and acquired disabilities tend to prioritise the restoration and development of physical function and capability. These policies include the United Nations Convention on the Rights of Persons with Disabilities [20] and the World Health Organization (WHO) Rehabilitation 2030 report [21]. These policies, while laying important groundwork for improving access to assistive technology, vocational rehabilitation and other practical interventions to promote social inclusion, largely neglect concerns related to psycho-emotional wellbeing, in particular the fostering of positive self-identities, social belonging and citizenship. Although restored ‘mental ability’ is mentioned in the UNCRPD as a goal of rehabilitation, this appears to refer to cognitive functioning rather than mental health and well-being. These international priority-setting documents influence local policies and practices in rehabilitation. For example, the South African White Paper on the Rights of Persons with Disabilities of 2015 [22] reflects this focus on restoring physical function and practical capability in rehabilitation, which in turn influences the funding, monitoring and evaluation of services for persons with disabilities [23].

Correspondingly, my qualitative work on VI rehabilitation services in South Africa found that these interventions largely prioritise practical skills, tools and techniques to adapt to life with VI. While service users found these practical interventions to restore and develop capability extremely valuable, they felt that their mental health was ignored and that, within prevailing, unequal power dynamics in rehabilitative organisations, their citizenship was undermined [24,25]. Empirical and autoethnographic work on VI and blindness shows that vision loss brings with it not only practical difficulties related to physical function, but psycho-emotional challenges related to a sense that one’s identity and place in the world has been permanently disrupted [4,26,24]. This sense of personal and social dislocation has in large part to do with encountering, in various spheres of life, a set of deeply embedded and negative sociocultural beliefs about blindness [4,6]. Consequently, newly blind persons must navigate not only the practical implications of vision loss, but also the psycho-emotional and social implications of having their nature, needs and capabilities interpreted through this negative framework of meanings, through which they also are now compelled to interpret themselves.

Therefore, alongside the practical interventions found in VI rehabilitation, newly blind persons need support to process difficult emotions and to develop positive self-identities based on embracing the differences of VI and exploring alternate ways to be and to participate in the world [27]. Yet, my research with newly blind people found that rehabilitation lacks formal psychological support, including professional support to assist with the processing of trauma, and space where service users are enabled to collectively voice experiences that may include loss, grief and struggle as a means to move into a new, secure and authentic sense of self and belonging [28]. Work in critical disability studies has suggested that rehabilitation practice tends to focus primarily on the provision of practical solutions, and is reluctant to engage with emotional and identity-related aspects of the experience of sight loss and blindness. This tendency is viewed as indicative of a corrective and normalising drive in society in general, and rehabilitation in particular, which invalidates diversity [4,27,17]. These scholars suggest that rehabilitation is a process aiming to support a compliant adjustment to an inequitable, inaccessible world, rather than a process where practical mastery can be developed alongside authentic and positive disability identities, involving the development of a secure sense of belonging and the self. Rehabilitation services that respond to the practical, psycho-emotional and social predicaments of disabled persons hold the potential to facilitate the emergence of empowered and active citizens with disabilities.

## 3. Social Inclusion and Invisibility (Heidi Lourens)

### An auto-ethnographic perspective on visual impairment, inequality and inclusion in South Africa

‘There is no greater agony than bearing an untold story inside you.’

– Maya Angelou [29]

Autoethnography is a mode of writing that uses the interpretation of experience to make sense of authors’ outer world (such as culture) and their inner world (like emotions, thoughts and behaviours) [30]. It therefore neatly embeds personal experiences within relationships, context and culture. This blend of gazing outward and inward makes autoethnography particularly suitable to narrate the disability experience [31]. Telling the story of disability requires an exploration of the external environment (including experiences of material inaccessibility, exclusion and stigma) while also unravelling the internal world (including the emotional impressions left by social and embodied experiences) [31].

Firstly, for disabled persons, living in a society created only to be ‘habitable’ to others is an elemental part of one’s life experience [32]. As just one example, easy access to transportation, and the empowerment and participation facilitated by freedom of movement, is denied to most visually impaired persons in our society. With public transport unavailable, unreliable and often unsafe, persons with VI often depend on the unpredictable goodwill of others to access this crucial resource [33], undermining their participation in education, employment and community life. Second, the history of disability around the world is one of silencing and concealment, meaning that the experiences of exclusion, prejudice and marginalisation suffered by the disability community are typically hidden. Social change, if it is to occur, relies on the breaking of this silence and telling stories of how mechanisms of inequality work in individual lives. Clear, articulate narratives of socially engendered struggle can bring to light the effects of an unjust material world on the inner lives of disabled persons, while also driving political mobilisation and solidarity.

Authentic disability-related stories of loss, trauma and grief are often dampened down or silenced, as such narratives may evoke a host of anxieties for the non-disabled world [34,28]. While the world of tangible barriers to participation for disabled persons is crucially important, an auto-ethnographic initiative holds that the ways in which those material realities shape inner experiences are equally worthy of investigation [15]. Also, the impressions left by lives of exclusion are all too real. Living in a world that was not designed with disabled persons in mind sends the subliminal message that they/us do not belong here. As I write elsewhere, ‘Every stare, every inaccessible book, every transport crisis contains the visceral message that I do not belong in this world; that this world is not my home [33].’

Keeping quiet about life in an inaccessible world and the difficult emotions and experiences it evokes will not only obscure and maintain the injustices of that world, but also undermine one’s emotional well-being. Hiding the effects of difficult experiences will in all likelihood transform these hidden parts into unacceptable and even shameful segments of the self [31]. Psychodynamic psychotherapists believe that free association — talking freely and unencumbered about experiences, feelings and thoughts, will aid in uncovering emotionally laden unconscious conflicts, thus leading to healing [35]. Similarly, since autoethnography requires introspection and free association, it may also contain therapeutic value for disabled persons.

## Conclusion

The profound discrimination and deprivation suffered by disabled persons around the world is a crisis we are only beginning to meaningfully address. Undoing lifelong exclusion requires examining how disablism is embedded in the very fabric of our societies — from material organisation, public administration and culture, to the community and relational levels. As with other forms of prejudice, it requires bringing to consciousness and un-learning layers of prejudiced assumptions about the nature and potential of disabled persons in which we have all been steeped, and from there, working to create social environments that are accessible and welcoming to all. Prejudiced beliefs surrounding VI are ancient and stubborn and require their own particular attention — ensuring that the potential of VI persons to flourish and contribute to the community will not be lost.

## Data Accessibility Statement

We hereby confirm that all authors have access to the data on which this publication is based.
